# Temporal Integration of Movement: The Time-Course of Motion Streaks Revealed by Masking

**DOI:** 10.1371/journal.pone.0028675

**Published:** 2011-12-20

**Authors:** David Alais, Deborah Apthorp, Anna Karmann, John Cass

**Affiliations:** 1 School of Psychology, University of Sydney, Sydney, New South Wales, Australia; 2 Universität Regensburg, Regensburg, Germany; 3 School of Psychology, University of Western Sydney, Sydney, New South Wales, Australia; Barrow Neurological Institute, United States of America

## Abstract

Temporal integration in the visual system causes fast-moving objects to leave oriented ‘motion streaks’ in their wake, which could be used to facilitate motion direction perception. Temporal integration is thought to occur over 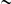
100 ms in early cortex, although this has never been tested for motion streaks. Here we compare the ability of fast-moving (‘streaky’) and slow-moving fields of dots to mask briefly flashed gratings either parallel or orthogonal to the motion trajectory. Gratings were presented at various asynchronies relative to motion onset (from 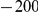 to 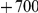 ms) to sample the time-course of the accumulating streaks. Predictions were that masking would be strongest for the fast parallel condition, and would be weak at early asynchronies and strengthen over time as integration rendered the translating dots more streaky and grating-like. The asynchrony where the masking function reached a plateau would correspond to the temporal integration period. As expected, fast-moving dots caused greater masking of parallel gratings than orthogonal gratings, and slow motion produced only modest masking of either grating orientation. Masking strength in the fast, parallel condition increased with time and reached a plateau after 77 ms, providing an estimate of the temporal integration period for mechanisms encoding motion streaks. Interestingly, the greater masking by fast motion of parallel compared with orthogonal gratings first reached significance at 48 ms *before* motion onset, indicating an effect of backward masking by motion streaks.

## Introduction

Visual perception may seem compellingly real and immediate, but it does not arise instantaneously. The neurons underlying our visual experience operate on an integrate-and-fire principle, and so it takes time for their response to build up. Within their integration period, information is accumulated, and the eventual response is a function of the summed inputs over that time span. There are advantages to integrating information over time. One obvious benefit is that a weak signal is more likely to be detected, as its sum over the integration period may exceed a neuron's threshold, even though the instantaneous signal may be weak and sub-threshold. This is particularly useful for detecting static spatial signals, as they effectively accumulate greater intensity over longer periods of integration. Temporal integration, which is thought to occur over about 100 ms in early visual cortical neurons [1]–[4], is one of the main reasons that we are still able to see when light levels are low or visual signals are faint.

Apart from its obvious advantages, there are disadvantages to temporal integration. For one, it restricts the temporal resolution of the neuron. Because all activity within the integration period is summed into a single response that is monotonically related to the summed activity, discrete stimulus events within the integration period are not distinguished by that neuron. The only factor that counts in determining the response is the summed input at the end of the integration period. This is known as Bloch's law [5]. It means, for example, that two brief signals of duration *t* are equivalent to a single signal of duration 2*t*. Another problem is that any movement of the stimulus during the integration period will lead to blurring of the summed image. On a small scale, this is a problem even when the stimulus is perfectly still, as microsaccades will cause a degree of blurring in the image. The problem is obviously exacerbated if the stimulus itself is in motion, as the moving stimulus will be smeared along the axis of motion. For example, a translating point source of light will produce a line when integrated over time.

Although temporal blurring is usually regarded as a negative consequence of temporal integration, it has recently been suggested that the visual system could exploit it as a useful direction cue in motion processing. Geisler [6] coined the term ‘motion streaks’ to describe the trail left by moving stimuli as a consequence of temporal integration in the visual system. The streaks only exist in the neural representation of the stimulus – not in the physical stimulus – but nonetheless may be useful. Geisler suggested a model in which the streak is detected by orientation-selective neurons and then combines with output from the motion system to improve directional acuity. A good deal of recent data from both psychophysics and neurophysiology supports this model [7]–[14]. It is now clear that motion streaks, although generally not perceived, do contribute to motion perception, and can interact with form processes.

**Figure 1:**
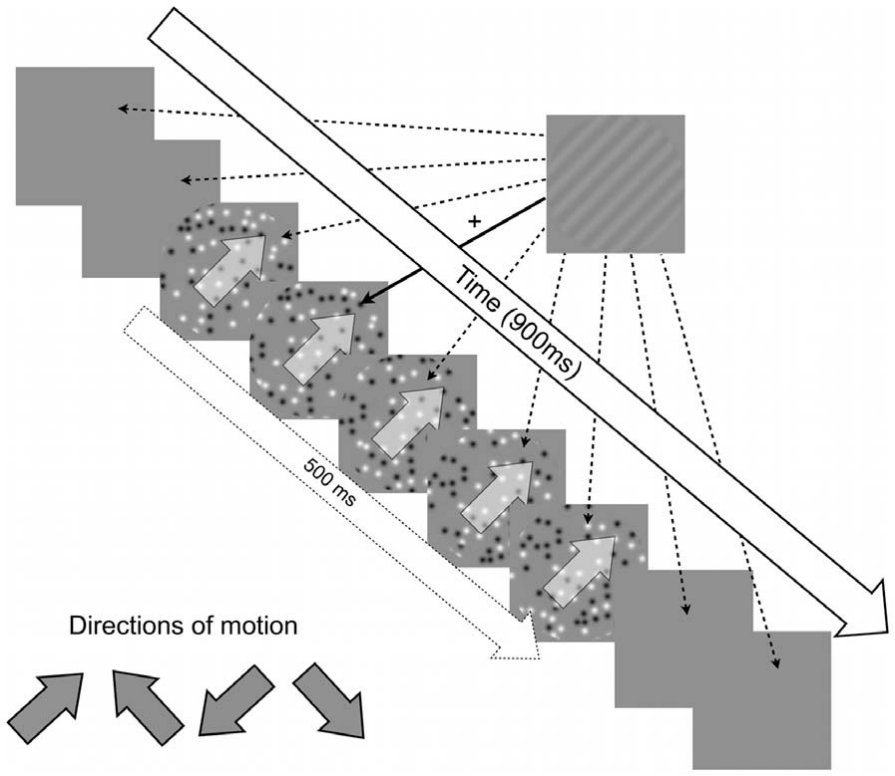
A schematic diagram of the temporal masking experiment. Probe orientation was defined relative to the direction of motion, either parallel with the motion trajectory or orthogonal to it. Four motion directions were randomly interleaved to help prevent adaptation. Participants' task was to judge whether the grating appeared in one of two motion displays, upper or lower. The upper and lower windows always contained the same motion direction on a given trial, and various test asynchronies from the full set of 21, ranging from 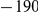 to 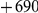 ms relative to motion onset, were randomly interleaved in blocks of trials.

To date, all of the published data relating to motion steaks has focused on the spatial or spatiotemporal domain. In this study, we address specifically the temporal domain. We will measure the temporal integration period for motion streaks by using fast-moving ‘streaky’ fields of dots to mask briefly flashed gratings, with the gratings presented at various asynchronies relative to motion onset (see Figure 1. At very short asynchronies, before the dots have translated far, there is little accumulated orientation information to mask the target grating and thresholds therefore should be low and close to unmasked baseline thresholds. As the asynchrony increases, however, the oriented streaks will lengthen and provide a more effective orientation mask, causing grating detection thresholds to rise. Thresholds should reach a plateau when the asynchrony matches the temporal integration period, as beyond this point the oriented streak information will not accumulate any further. We predict that masking will be strongest for fast motion parallel to the target grating, as fast motion will leave long motion streaks that will effectively mask the orientation of the parallel target grating.

## Results

### Masking threshold elevations

**Figure 2:**
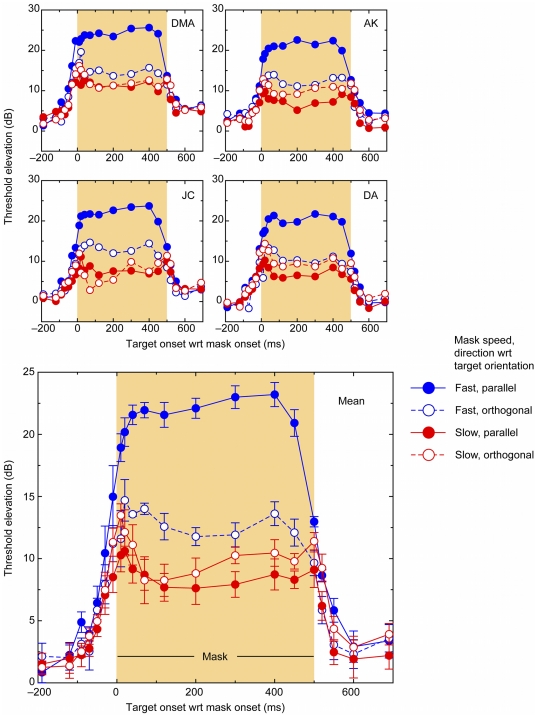
Masking elevations of a brief target grating from unmasked baseline as a function of presentation time relative to the onset (0 ms) of a 500 ms motion mask. The plot shows the means of four observers, plotted with 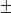
1 standard error bars. To capture any effects of backward and forward masking, target gratings were presented as early as 190 ms before the motion mask began (indicated by negative timing), as well as up to 190 ms after the motion mask ended. Results are shown for fast and slow motion masks, and for target gratings parallel and orthogonal to the motion direction.

Masking threshold elevations (individual results and group means, with 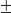
1 standard error bars) for all four conditions (slow/fast motion mask
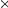
parallel/orthogonal target grating), are plotted in Figure 2 as a function of the asynchrony between the target and the onset of the motion mask. Threshold elevation is measured in decibels, as per Equation 1:
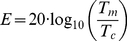
(1)where *E* is threshold elevation, *T* 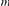 is the masked threshold, and *T* 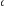 is the unmasked threshold.

For negative asynchronies (indicating the grating was presented prior to the motion mask) all conditions produce broadly similar levels of masking. Masking levels also appear similar across conditions for asynchronies greater than 500 ms (indicating the grating was presented after the motion mask). Importantly, the masking functions diverge during the period when the motion mask is present, with masking being strongest in the fast parallel condition. This effect confirms our prediction, and is consistent with the presence of long motion streaks interfering with the detection of an iso-oriented target grating. Consistent with our reasoning that this effect is due to iso-orientation masking, when the target grating was oriented orthogonally to the motion streaks (fast orthogonal condition), masking was greatly attenuated, by about 8–10 dB.

**Figure 3:**
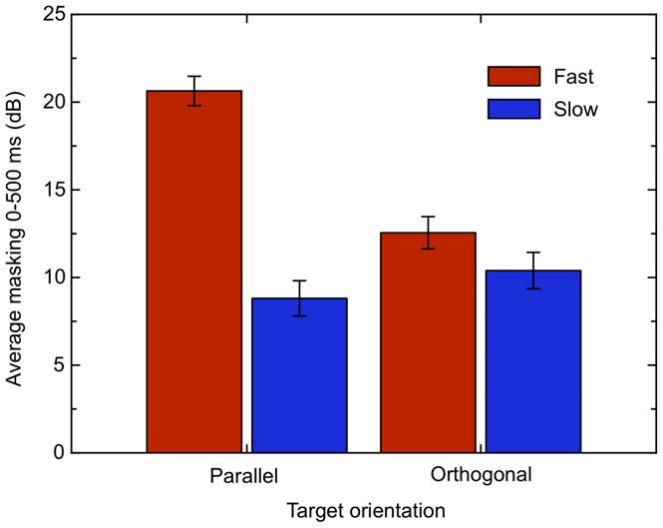
Results from a repeated-measures one-way ANOVA analysis of the averaged threshold elevations for each condition during the masking period. Error bars show 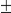
1 standard error.

To confirm these masking effects among the four conditions, the group mean data were analyzed in a two-way repeated-measures ANOVA. To do this, we first averaged the threshold elevations within each of the four conditions during the period when the mask was present (i.e., asynchronies from 0–500 ms). The ANOVA showed significant main effects of speed, *F*(1,3) = 126.2, p = 0.002, and orientation, *F*(1,3) = 49.9, p = 0.006, and, more importantly, a significant interaction between speed and orientation, *F*(1,3) = 126.8, p = 0.002 (see Figure 3). All conditions produced threshold elevations significantly greater than 0 (see Table 1). Pairwise comparisons between the slow motion masking conditions showed these were not significantly different from each other, *t*(3) = 1.85, p = 0.16, and the two orthogonal conditions (fast vs. slow) also did not differ, *t*(3) = 2.11, p = .13. However, fast parallel masking was significantly greater than fast orthogonal, *t*(3) = 32.99. p
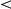
0.001, and also than slow parallel, *t*(3) = 38.07, p
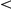
0.001.

**Table 1:** T-values for single-sample t-tests comparing threshold elevations to a test value of 0 for each of the masking conditions, averaging across four subjects.

	Parallel	Orthogonal
Fast	24.63 (0.001)	13.62 (0.004)
Slow	8.76 (0.012)	10.02 (0.008)

P-values are in brackets. Tests were two-tailed, and p-values are Bonferroni-corrected for multiple comparisons.

### Masking time-course

**Figure 4:**
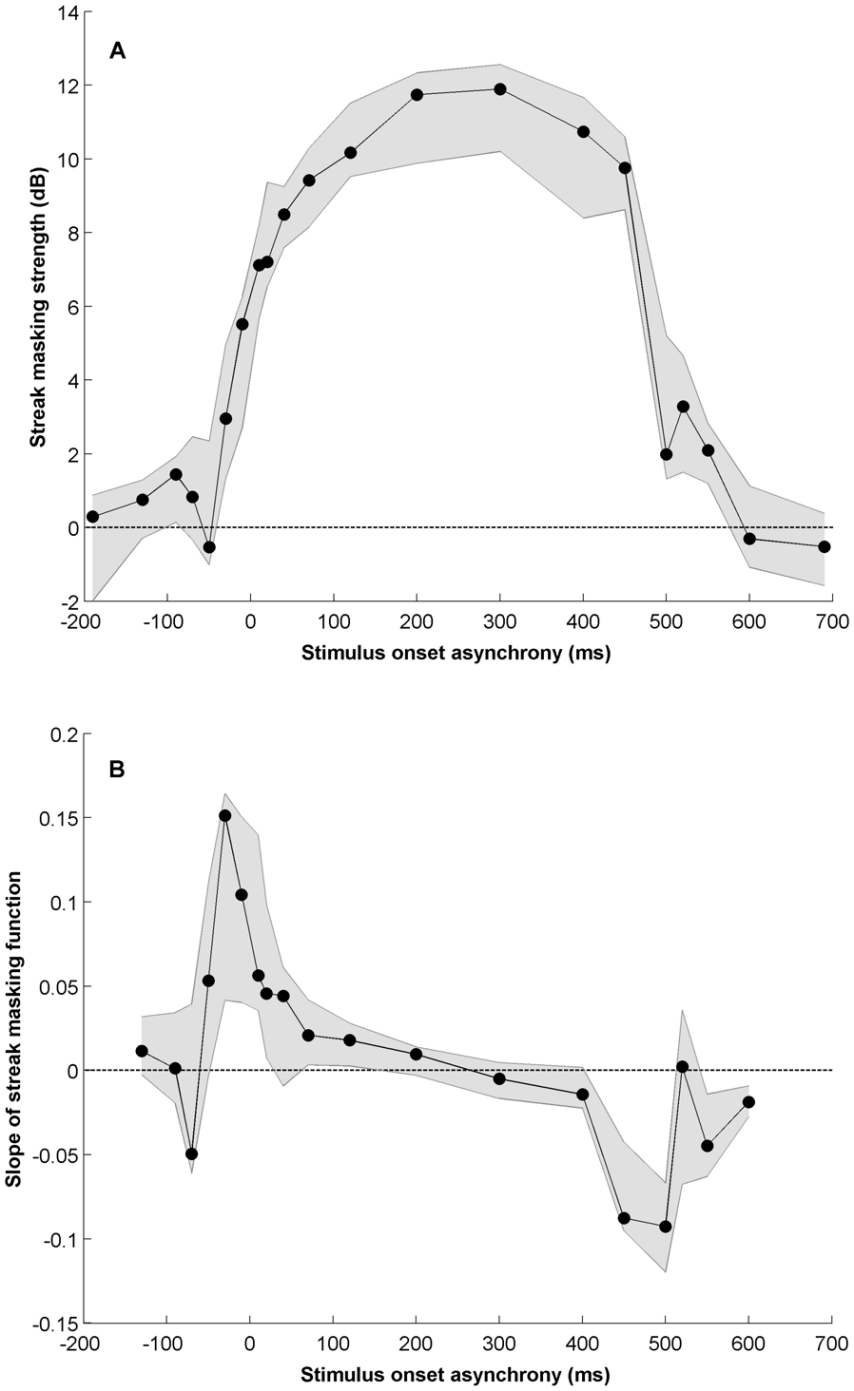
Masking specific to motion streaks – differences and first derivative. a) The masking component specific to motion streaks plotted as a function of the grating target's asynchrony relative to motion onset. The plot shows group means (n = 7), flanked by 90% confidence intervals, of the difference between the fast parallel and fast orthogonal conditions. This contrast reveals the streak-specific masking component because while both conditions contain fast translating dots (and therefore motion streaks), masking occurs only in the parallel condition where target and mask are iso-oriented. b) The first derivative of the streak-specific masking component, calculated using the three-point method, plotted in panel A, flanked by 90% confidence intervals. The sustained increase in masking in panel A around 0 ms is indicated by the four consecutive positive slopes around 0 ms. The points either side of this series of four points are not significantly different from zero. Linearly interpolating between the lower confidence intervals, this elevated series of points is significant between 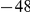 and 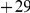 ms, indicating a temporal integration period of 77 ms.

The main aim of this experiment was to demonstrate an accrual of masking over time that was both orientation- and speed-dependent. The dependencies on orientation and speed are clear from the masking functions in Figure 2 and are confirmed in the ANOVA: masking is strongest for fast motion masks and parallel target gratings. The key contrast to reveal the effect of motion streaks is the difference between the fast parallel and the fast orthogonal conditions. Figure 4a shows this contrast for the group mean data for seven subjects; any effect of masking significantly greater than zero can be attributed to the presence of motion streaks aligned with the target grating. To determine significance, we used a bootstrapping procedure to define 90% confidence intervals, plotted here as the gray shaded area flanking the data points. This procedure involved resampling the data of each subject 2000 times to obtain a population distribution of bootstrapped means for each subject. Then, for each iteration, the four bootstrapped means from each subject were averaged into a group mean, producing a population of 2000 group means. These were then ranked and the means bounding the central 90% of the population defined the confidence limits. This allows easy directional tests of significance as any masking elevation where the lower confidence limit exceeds zero is significant at the 0.05 level.

The two distinct aspects of the streak-dependent masking function (formed by subtracting fast orthogonal from fast parallel conditions) in Figure 4a are the steep rise in masking around motion onset (0 ms) and the steep fall around motion offset. Between the rise and fall there is a period of sustained masking throughout the motion mask period. We are primarily concerned with the steep rise in masking around motion onset attributable to the increasing elongation of streaks throughout the period of temporal integration. Our rationale was that this rise in masking would saturate at a point corresponding to the temporal integration limit for motion streaks.

To determine the point at which the rise in masking reaches a plateau, we took the first derivative of the streak-specific masking function, as shown in Figure 4b. This analysis reveals the changes in slope across the masking function and allows us to define the plateau point as the target asynchrony at which the steeply rising section around 0 ms reduces to a slope not significantly different from zero. Similarly, we can easily find the beginning of the steep rise in masking by determining the asynchrony where the slope first increases significantly above zero. The slopes plotted in Figure 4b are flanked by 90% confidence intervals (generated by bootstrapping), so that points with a lower confidence limit above zero are significantly above zero at the 0.05 level.

Using this approach, we determined the period of temporal integration implied by the steep initial rise in masking. In Figure 4b, the series of significantly positive slopes centered around motion onset begins between the 3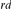 and 4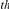 points of the function. By linearly interpolating between the lower confidence intervals, we find that the point where the positive slope first rises significantly above zero is at 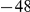 ms. The same interpolation procedure shows that the positive slope decreases to a value not significantly different to zero between the 8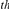 and 9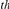 points at 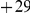 ms. Together, this range defines a temporal integration period of 77 ms. By applying the same analysis at the end of the motion mask period (to the upper confidence intervals), the period of rapidly declining masking indicated by negative slopes ranged from 402 ms to 506 ms, a period of 104 ms.

## Discussion

### Masking data

The important point for our purposes is that there is a strong interaction between speed and orientation. The orientation effect for the fast ‘streaky’ motion mask is large, with target grating thresholds showing 10 dB more masking when they are oriented parallel with the direction of motion than when oriented orthogonally. This is the critical comparison for our streak hypothesis, as streaks should only be present in fast motion masks, and the masking effect should only occur when the test grating is parallel to the streaks. There is no orientation effect of masking at low speeds, consistent with there being no oriented content in the slow motion dots. This agrees with our previous data and those of Geisler [6] that the slow motion speed we chose is below the threshold for producing motion streaks.

We had expected that there would be an elevation of the fast orthogonal condition over both slow motion conditions because high temporal frequencies suppress low temporal frequencies and static stimuli such as the test grating, and do so across all orientations [15]–[19]. Even low temporal frequency patterns that are well above threshold can be rendered invisible by this process (examples include motion-induced blindness [20], [21] and adaptation-induced blindness [22]). Although the fast orthogonal condition appears to be consistently higher than the slow conditions, this difference did not reach significance.

An interesting point to note is that there is a considerable unoriented component to masking (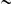
10 dB) which does not appear to be tuned for orientation or speed (see Figures 2 and 3 and Table 1). This is consistent with previous findings on masking of gratings by moving stimuli [7], [23]. The mechanism for this is not clear, although it may relate to cross-orientation suppression [24]–[29], which is known to be isotropic (i.e., not tuned for orientation). Interestingly, though, there is little temporal-frequency tuning evident in the unoriented masking seen here: the difference between fast and slow orthogonal conditions is not significant.

### Time course of motion streak masking

Streak-dependent masking strength increased over a period of 77 ms: this provides an estimate of the temporal integration period for motion streaks. Geisler assumed an integration period of 100 ms when calculating his critical blob speed [6], and other authors using psychophysical procedures have found similar estimates [4], [30], [31]. Our estimate of the temporal integration period of 77 ms is somewhat shorter than these estimates. There are a couple of important differences between these studies and our own that could potentially explain this difference: one concerns the luminance of the stimulus display and the other is methodological. To take the first point, the stimuli used in Geisler's study had a much lower mean luminance than ours (1.36 cd/m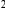 vs. 33.7 cd/m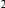, a difference of 1.4 log units), and it is known that the temporal integration period is longer at low luminance. Indeed, the integration period declines log-linearly as a function of illumination by roughly 20 ms per log unit from about 100 ms at 0 log Trolands to about 25 ms at 4 log Trolands [32], a rate that squares with our observation of a 75 ms integration period at high luminance and Geisler's assumed 100 ms period at a luminance 1.4 log units lower. Note however that most psychophysical experiments are done at luminances similar to ours, and vision in real-world contexts usually contains luminances that are at least this high, and so our slightly shorter estimate of the temporal integration period is probably more appropriate in most cases.

The second important difference between our study and others that have estimated temporal integration is methodological. Traditionally, the temporal integration limit was estimated using a threshold-versus-duration function. As embodied in Bloch's law [5], the threshold-versus-duration function initially shows a linearly declining detection threshold as stimulus duration increases until an elbow is reached where the function flattens out to constant zero slope (or a slightly negative one) for further increases in duration. Traditionally, psychophysical studies estimating temporal integration periods have used threshold-versus-duration functions and taken the elbow point to define the limit of integration, the point at which all available signal has been accumulated and longer stimulus durations cannot further improve performance. Snowden and Braddick [4] found the ‘elbow’ in this function for motion stimuli to occur at 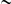
100 ms. However, this method may over-estimate the integration period. The reason is that in any noisy system, as stimulus duration increases, probability summation over time will improve the likelihood of detection, and thereby continue to produce declining thresholds beyond the physiological temporal limit [33]. This confounding effect makes it difficult to define the elbow point of the threshold-versus-duration and therefore the temporal integration period itself.

An alternative method which eliminates the effect of probability summation employs a ‘two flash’ paradigm of constant total stimulus duration to estimate the temporal impulse response function of a linear filter. The temporal impulse response function describes the activity of a filter over time in response to a single pulse of input. This approach, developed within the framework of linear systems theory, assumes an initial linear filter followed by non-linear threshold mechanism which is triggered once a given activity level is exceeded. Because the filter is linear, knowing its temporal impulse response function allows its output to any arbitrary input to be found by summing the convolution of the stimulus at each instant with the response function. In the two-flash paradigm, the interval between the brief flashes is varied but the total stimulus duration remains constant, thereby controlling probability summation. These studies reveal two types of impulse response function: a ‘sustained’ response which is monophasic, and a ‘transient’ response which is biphasic [34]. The appropriate function to characterize the build-up of motion streaks from our translating Gaussian blobs is the sustained function, since the orientation-tuned units detecting the streaks exhibit this kind of response [31], [35]. Estimates of the sustained impulse response function suggest it has a half-width of about 40 ms [34], [36], which agrees well with the full-width estimates from the present streak masking study of 77 ms. Note that the full-width of the sustained temporal impulse response function is in effect a temporal integration estimate because the threshold mechanism can be triggered at any moment during the temporal impulse response period.

**Figure 5:**
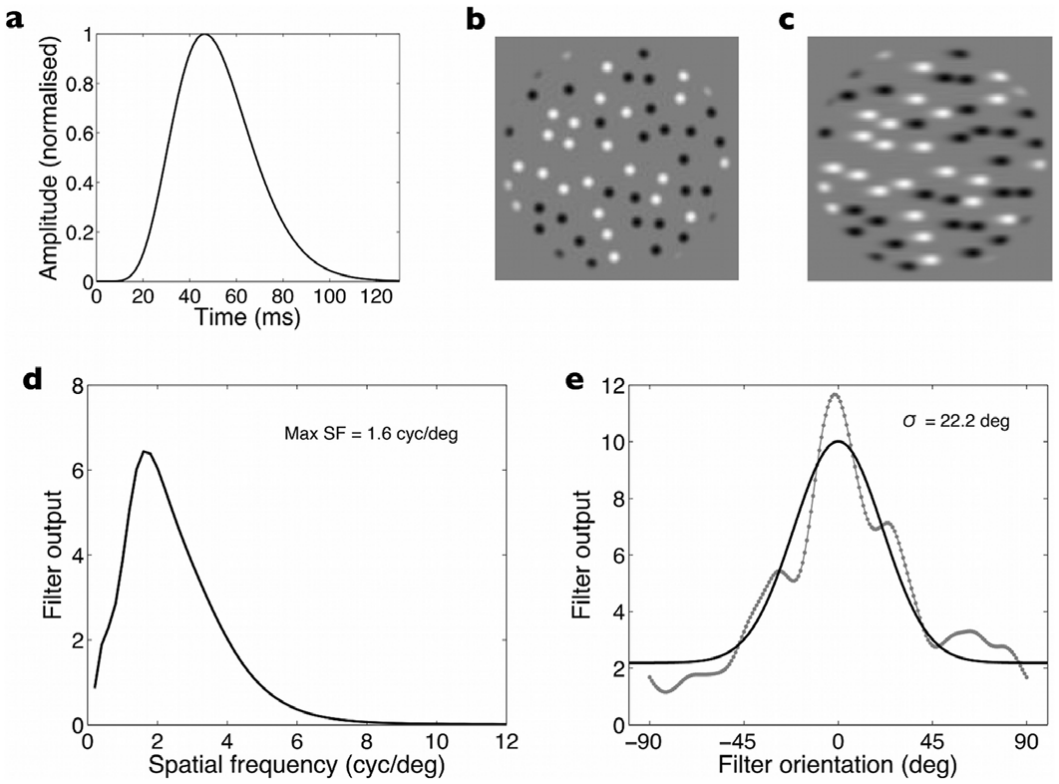
a) A sustained impulse response function defined by Manahilov et al's (2003) equation (see equation 1, main text). The function plotted here has the following parameters: 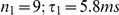 – the values Manahilov et al found best described sustained impulse responses at a frequency of 2 cyc/deg. At half-height, the impulse response has a full width of 41 ms. b) A single frame taken from the sequence of frames defining the fast translating blobs. c) The temporally smeared version of the blob stimulus that results from passing the fast translating blob image in panel b through the temporal impulse shown in panel a (i.e., performing a convolution integral). The output shown in panel c is the ‘streaky’ image that can be assumed to emerge following a simple linear filtering stage characterized by a sustained impulse response. d) The spatial tuning of the streak image in panel c. The figure shows the output of a sliding log Gabor filter computing the spatial energy at each spatial frequency from the minimum frequency to 12 cyc/deg in the direction orthogonal to the streaky elongations (i.e., vertically, in this case). The log Gabor had a spatial bandwidth of 1 octave and a narrow orientation bandwidth (1) oriented to sample vertically across the image shown in panel c. The peak frequency occurs at 1.6 cyc/deg and falls to half-height at 3.3 cyc/deg. e) The orientation tuning of the image in panel c at peak frequency. The data were obtained by rotating the log Gabor filter (1 octave spatial bandwidth, peak at 1.6 cyc/deg) in one-degree steps. Grey symbols show the filter output and the black line is the best-fitting Gaussian function (standard deviation = 22.2).

In Figure 5a we show a sustained impulse response function plotted using an equation taken from Manahilov et al. [36] and using the parameters they found best described the impulse response function for a spatial frequency of 2 cyc/deg. This frequency is appropriate to our stimuli because we have previously shown that our streak stimuli have a peak spatial frequency near 2 cyc/deg [23]. The model consists of a linear temporal filter, which has a temporal impulse response with excitatory and inhibitory components, each approximated by a cascaded low-pass leaky integrator and is described by the following equation:
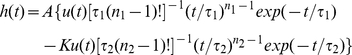
(2)where 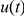 is the unit step function, 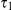 and 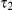 are the time constants of the two components, 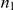 and 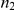 are the number of the cascaded low-pass stages of each component, 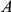 is a sensitivity factor and 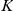 is a transience factor. In our case we are dealing with a sustained impulse response and so the transience factor is set to zero and the equation simplifies to a single-phase impulse characterized by the parameters of 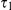 and 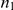, as shown in Figure 5a. The function plotted in Figure 5a has parameters 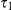 = 5.8 ms and 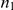 = 9, taken from Manahilov et al's Table 1.

The key point to note is that this impulse response function has a half-width of about 40 ms, and a significant overall elevation spanning about 70 ms or so. The sustained temporal impulse response function is therefore considerably shorter than psychophysical estimates of the temporal integration period of around 100 ms [4] and closer to the estimate we obtained here of 77 ms. In Figure 5c, we show the result of convolving the fast translating stimulus with the impulse response function shown in panel A to produce an image of the linear filter's output to the translating dots (i.e., the neural streak image). The resulting streak stimulus is temporally smeared along the axis of motion and we then Fourier analysed it to reveal its spatial properties. First, the Fourier amplitude spectrum was filtered using a sliding log Gabor filter. The filter had a 1-octave spatial bandwidth and was oriented orthogonally to the steaks with a narrow orientation bandwidth (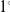). The filter's peak was shifted successively from the minimum to the maximum frequency in the amplitude spectrum to obtain the distribution of streak energy across spatial frequency. As shown in Figure 5d, the streak image exhibits a narrow spatial passband (across the elongations) peaking at 1.6 cyc/deg. Second, using the log Gabor filter with a peak at 1.6 cyc/deg and a 1-octave bandwidth, we rotated the filter around the Fourier amplitude spectrum in 1-degree steps so obtain the steak orientation tuning. As shown in Figure 5e, the orientation tuning is approximately Gaussian in shape, with the best-fitting Gaussian having a standard deviation of 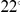. Given these orientation and spatial frequency characteristics of the streak stimulus, it is not surprising that it provided an appropriate mask for the target grating used in the experiment reported here which was iso-oriented with streaks and had a very similar spatial frequency of 1.54 cyc/deg.

### Adaptation, and forward and backward masking

In Figure 2 there appears to be a gradual rise in masking strength of about 3 dB for fast, parallel motion that occurs after the initial steep rise around the time of motion onset. It is likely that this gradual rise in masking is a consequence of orientation adaptation. In the fast parallel condition, where there are elongated motion streaks present in the motion mask, there would be a gradual accrual of orientation adaptation throughout the period of the motion mask that would not be present in the other conditions. Adaptation is well known to raise contrast thresholds, and can do so following very brief exposure to the adaptor such as the 500 ms motion stimulus used here [37]–[39], provided the test stimulus is brief (as ours was). By adapting the orientation channel that is used to detect the target grating, contrast detection thresholds would be expected to rise, and to do so increasingly as the target asynchrony increases from 0 to 500 ms. For this reason, we believe the gradual rise in masking after the steep initial onset in the fast parallel condition can be attributed to a modest adaptation effect.

The data in Figure 4a exhibit features that square well with what is known about forward and backward masking obtained using other paradigms [40]–[43]. Apart from the broad central band of threshold elevation coinciding with the motion mask (0–500 ms), there are small shoulders of threshold elevation in Figure 4a just prior to motion onset, and just after motion offset. The narrow band of threshold elevation for targets presented prior to the motion mask can be ascribed to backward masking [40]. Backward masking can occur for a rather wide range of stimulus onset asynchronies but has been found to be strongest for target onsets about 80 to 100 ms before mask onset [44] (see Figure 2a), consistent with the peak observed in our data at 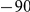 ms. There is also a narrow band of threshold elevation for targets presented after the motion mask, which is consistent with forward masking [45]. Forward masking occurs for a narrower timing range, being most effective for targets presented between 5 and 35 ms after mask offset [44] (see Figure 2f), with a peak effect at an of ISI 20 ms. These observations are very consistent with our data, which show a peak forward masking effect at 520 ms–20 ms after the offset of the motion mask. Neurophysiological recordings [46], [47] strongly suggested that forward and backward masking are related to suppression of, respectively, transients associated with target onset and target offset, a suggestion subsequently confirmed in recordings from awake behaving primates [48].

Masking may not only explain the shoulders of elevation shortly before and after the onset of the motion mask, it may also explain the slight rise and fall in masking strength over the course of the motion mask period. Geremek and Spillman [49] conducted a study of masking that varied the spatial configurations between a target and an adjacent mask. Although the spatial focus of this paper is very different to our own, a key similarity was that they manipulated mask duration while keeping the target duration constant. This manipulation demonstrated that masking strength increased with masker duration up to two to three hundred milliseconds, consistent with earlier nuerophysiological data by Macknik and Livingstone [44]. A similar pattern can be seen in our own data. In Figure 4a, starting at the offset of the motion mask (i.e., at 500 ms, where there is no post-target mask) and moving to the left (so that the duration of the post-target masking period increases), the streak masking effect increases for about two hundred milliseconds or so beyond which it would be expected to stabilize for any further increases in masking duration. The fact that the streak masking effect also rises from the beginning of the motion mask period (i.e., 0 ms and greater) must be due to another factor. One possibility is that it is due to an increase in streak adaptation over the mask period raising thresholds for the parallel target grating, as noted above. Another possibility is that it is simply due to a trade-off between forward masking early in the mask period and backward masking later in the mask period. Indeed, a very similar rise-and-fall pattern of data can be seen in Macknik and Martinez-Conde's [50] masking data (see Figure 8) for a brief target presented within a mask period of 300 ms (not so different to our mask duration of 500 ms).

Turning to Figure 4b, it is interesting that significant masking by fast parallel motion was evident 48 ms before mask onset, indicating an effect of backward masking by motion streaks. Although there is a general increase in detection thresholds in all conditions just before mask onset (see Figure 2), the pre-mask elevation in Figure 4b reveals a specifically orientation- and speed-dependent (i.e., streak related) masking effect. It may seem surprising that the streak information, which takes time to accumulate, can effectively mask stimuli presented before motion onset. However, previous studies on backward masking of static grating stimuli [51] have shown that the time course of backward masking is strongly orientation-dependent so that iso-oriented targets and masks (such as the streak masking of gratings used here) produce much stronger and earlier masking effects than cross-oriented masks.

The estimate of the temporal integration period at offset (104 ms) is considerably longer than the integration period seen at onset (77 ms). In principle, the masking function at onset should conform to the integral of the temporal impulse response function, and the masking function at offset should be the mirror reversal of the integral. However, there are at least two factors that may contribute to the observed asymmetry. The first factor is the adaptation effect noted above, which may delay the masking function's return to baseline (broadening the apparent integration period at offset). The second factor is forward masking. It is clear from Figure 4a that thresholds remain significantly elevated for the two points immediately following the offset of the motion mask. This would also delay the masking function's return to baseline and broaden the estimated integration period at offset.

To summarize our findings, we have used motion streaks to mask iso-oriented grating targets presented at various onset asynchronies. In doing so, we have demonstrated the time course of motion streaks. We show that motion streaks accrue over a period of about 77 ms, gaining strength as maskers over this period, before leveling off for longer asynchronies. We attribute this time period to the temporal extent of a sustained impulse response in early linear filters and show that our estimated streak period agrees with recent modeling of temporal impulse response functions. The effect of the sustained temporal response on a translating dot pattern is to smear it along the direction of translation, producing an elongated “streaky” image with a tight spatial and orientation tuning. The period of accrual of motion streaks estimated by our masking approach (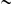
77 ms) is slightly shorter than suggested by earlier temporal integration studies. However, these earlier studies used the threshold-versus-time approach which may have slightly overestimated the period of temporal integration in early visual cortex. We believe the linear filter model is more parsimonious approach to estimating temporal integration.

## Materials and Methods

### Participants

Participants were four experienced psychophysical observers, all of whom had normal or corrected-to-normal vision. An additional three experienced observers, naïve to the purpose of the experiment, participated in the fast parallel and orthogonal conditions for the main time-course analysis.

### Ethics statement

This research was approved by the University of Sydney's Human Ethics Research Committee (project no. 10186). All subjects participated voluntarily and gave informed written consent.

### Apparatus and stimuli

Stimuli were programmed in Matlab version 7.4 using the Psychophysics Toolbox [52], [53]. Participants viewed the stimuli from a distance of 57 cm on a Mitsubishi DiamondView 22-inch CRT monitor with a screen resolution set to 1024
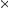
768 pixels and a vertical refresh rate of 100 Hz, controlled by a MacPro computer with a dual-core Intel Xeon processor. A Cambridge Research Systems Bits++ digital-to-analogue converter was used to provide 14-bit resolution in order to enable precise measurement of low contrast thresholds. The monitor was gamma-corrected in software to achieve linearity of output.

The mask stimulus was a drifting random dot display of 500 ms duration, with each frame composed of 80 Gaussian blobs with a standard deviation (SD) of 0.08 degrees, giving a dot diameter (defined as 4× dot SD) of 0.32 degrees. Half of the dots were dark and half were light, drifting with 100% coherence on a mid-grey background. Maximum and minimum dot luminances were 67.3 and 0.26 cd/m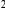 and background luminance was 33.7 cd/m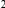. The dots drifted with a speed controlled by manipulating the pixel step size on each video frame. Two speeds were compared, a fast speed of 13.02/s that is well above Geisler's critical streak speed of one dot-width per 100 ms [6], and a slow speed of 1.63/s, that is well below (note that we have also empirically tested these speeds; [9]). The initial position of each dot was randomly determined and all dots wrapped around the aperture. Motion stimuli (the ‘mask’) were presented within virtual apertures 4.88 in diameter that were centered on points located 3.81 above and below a white fixation cross (see Figure 1). During the test phase, the fixation cross changed to black and the ‘target’ stimulus (a low-contrast sine wave grating) appeared in either in the upper or lower test aperture.

The target stimulus was a grating briefly presented either parallel or orthogonal to the direction of motion at various onset asynchronies relative to the onset of the motion mask. The target grating was shown for three video frames with the contrast of each frame sampled from a Gaussian temporal profile that was centered on the middle frame and had a standard deviation of 10 ms. The grating had a spatial frequency of 1.54 cyc/deg which was chosen because it approximately matched the spatial scale of the streaks left by the dark and light blobs (see Figure 1, which shows a scaled version of the on-screen stimuli). The target asynchronies relative to motion onset ranged from 200 ms before the motion to 700 ms after motion onset. Note that as the motion mask lasted 500 ms, the final asynchronies tested points after motion offset. A total of 21 asynchronies were tested, from 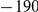 to 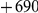 ms.

### Procedure

Conditions were blocked by speed (fast or slow mask) and orientation (parallel or orthogonal) and stimuli were viewed binocularly. For a given condition of trials (e.g., slow orthogonal), a subset of seven of the 21 target onset asynchronies was chosen, and during that block, the masking motion was randomly interleaved among four directions (45, 135, 225 and 315) to minimize motion adaptation affecting the results. In a spatial two-interval, two-alternative forced-choice task, the subject had to indicate whether the grating appeared in the upper or lower aperture, and contrast thresholds for grating detection were determined using QUEST adaptive staircases [54], modified to use cumulative Gaussian psychometric functions and converge on a threshold value of 75% correct performance. In a given block, one QUEST was used for each of the target onset asynchronies, and three runs of each block were undertaken. The data from the three QUESTs for each asynchrony were pooled and fitted with a cumulative Gaussian psychometric function, the mean of which defined the target detection threshold for that asynchrony. This procedure was repeated for the remaining two subsets of target onset asynchrony, which completed all measurements for a given condition (e.g., slow orthogonal). The remaining three conditions were tested in the same manner. All conditions, and subsets of target asynchronies, and the three repetitions of those, were all conducted in randomized order. Detection thresholds were also made for grating targets without the presence of masking motion to provide an unmasked baseline. The dependent variable was threshold elevation due to masking: that is, the target detection threshold in the masked conditions divided by the threshold in the unmasked control condition, expressed in decibels, as in Equation 1.
